# Synthesis of nonionic-anionic colloidal systems based on alkaline and ammonium β-nonylphenol polyethyleneoxy (n = 3-20) propionates/dodecylbenzenesulfonates with prospects for food hygiene

**DOI:** 10.1186/1752-153X-6-95

**Published:** 2012-09-08

**Authors:** Calin Jianu

**Affiliations:** 1 Department of Food Science, Faculty of Food Processing Technology, Banat’s University of Agricultural Sciences and Veterinary Medicine, Calea Aradului 119, 300645, Timisoara, Romania

**Keywords:** Polyoxyalkylene ether acid with higher alkylaryl group, Alkylaryl polyethoxy carboxylate surfactants, Ethoxylated nonylphenol carboxylates, Carboxy propylated nonionic alkylaryl surfactants, High alkylphenol polyether carboxylic acid salts

## Abstract

**Background:**

The main objective of this work was to obtain a binary system of surface-active components (nonionic soap – alkaline and/or ammonium dodecylbenzenesulfonate) with potential competences in food hygiene, by accessing a scheme of classical reactions (cyanoethylation, total acid hydrolysis and stoichiometric neutralization with inorganic alkaline and/or organic ammonium bases) adapted to heterogeneously polyethoxylated nonylphenols (n = 3-20). In the processing system mentioned, dodecylbenzenesulfonic acid, initially the acid catalyst for the exhaustive hydrolysis of β-nonylphenolpolyethyleneoxy (n = 3-20) propionitriles, becomes together with the nonionic soap formed the second surface-active component of the binary system.

**Results:**

In the reaction scheme adopted the influence of the main operating (duration, temperature, molar ratio of reagents) and structural parameters (degree of oligomerization of the polyoxyethylene chain) on the processing yields for the synthetic steps was followed. The favorable role of the polyoxyethylene chain size is remarked, through its specific conformation and its alkaline cations sequestration competences on the yields of cyanoethylation, but also the beneficial influence of phase-transfer catalysts in the total acid hydrolysis step. The chemical stability of dodecylbenzenesulfonic acid (DBSH) at the temperature and strongly acidic pH of the reaction environment is confirmed. The controlled change of the amount of DBSH in the final binary system will later confer it potential colloidal competences in food hygiene receipts.

**Conclusions:**

The preliminary synthetic tests performed confirmed the prospect of obtaining a broad range of useful colloidal competences in various food hygiene scenarios.

## Background

Good household hygiene [1-6] is important throughout the world for preventing infectious diseases. For additional protection, manufacturers are developing antibacterial products specifically for industrial and home use. These products are not true disinfectants; but they do decrease the number of living microorganisms on skin or surfaces to significantly lower levels.

Among the new generation of antibacterial household products that have recently appeared on the market, hand dishwashing liquids have become increasingly popular. They are classical dishwashing liquids based on anionic and nonionic surfactants, to which one or more antibacterial agents have been added. These formulae have been optimized to maintain their cleaning/degreasing performance and to fight bacteria on hands, in the washing solution, and on washing implements. In the United States, these products are regulated by either the EPA (Environmental Protection Agency) or the FDA (Food and Drug Administration), depending on specific claims.

Sanitation within the food industry means the adequate treatment of food-contact surfaces by a process that is effective in destroying vegetative cells of microorganisms of public health significance, and in substantially reducing the numbers of other undesirable microorganisms, but without adversely affecting the food or its safety for the consumer (FDA, Code of Federal Regulations (CFR), 21CFR110, USA). Sanitation Standard Operating Procedures are mandatory for food industries in US, which are regulated by 9 CFR part 416 in conjunction with 21 CFR part 178.1010. Similarly, in Japan and Europe, food hygiene has to be achieved through compliance with food sanitation laws.

**Figure 1:**
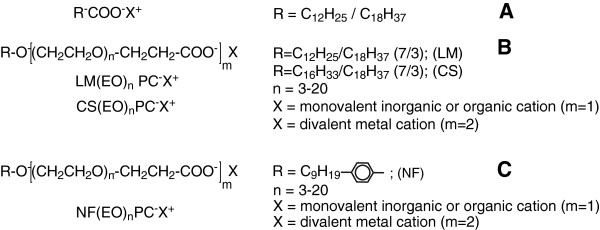
Structures of traditional and nonionic soaps.

Traditional soaps (Figure 1A) (alkaline salts of long-chain acids) have been known as structures and practical importance (sanitation agents) for hundreds of years since the discovery of the alkaline hydrolysis process (saponification of fats and oils). The two major drawbacks of classical soaps are their low solubility in aqueous solutions (opalescence) and their high sensitivity to the hardness of waters used in sanitation processes due to the low solubility of their calcium and magnesium salts.

Nonionic soaps (Figure 1B and 1C) are known in specialty literature [7-14] and food technology practice as colloid structures with surface-active competences superior to those of traditional soaps, due to the presence within the molecular ensemble of heterogeneous polyoxyethylene chains (n = 3-20).

The soaps are probably the oldest surfactants that have been used as washing and cleaning agents. Washing powders in Western Europe contain a certain percentage of soap of higher fatty acids, which form calcium soaps in the washing flote and thus act as defoaming agents. Ethoxylates can be transformed into polyether carboxylates by selective derivatization of terminal primary hydroxyl groups [carboxymethylation of the hydroxyl group with sodium chloroacetate (nonionic soaps)].

Normally, the degree of the ethoxylation (oligomerization degree of ethylene oxide in the polyoxyethylene chain) (n) is between 2 and 20(50) units ethylene oxide. The sodium salts of polyether carboxylic acids are water soluble, mild, and very resistant against water hardness and have extremely good dispersal and emulsifying power.

Further variations can be achieved by using:

▪  propylene oxide/ethylene oxide adducts (copolymers) instead of simple ethoxylates for carboxyalkylation

▪  alkylphenols (octyl-, nonylphenol) instead of fatty alcohols

▪  carboxyalkylation (C_1_-C_4_) instead of carboxymethylation only

1939 is widely accepted in the specialty literature [15] as the year of the first report of nonionic soaps, through the patent granted to H. Haussmann at I.G. Farben Industrie.

It was a natural consequence, on the one hand, of the scarcity of fatty materials which began to manifest ever more acutely in Europe at the beginning of World War II, but also of the poor quality of waters used in the technological processes (excessive hardness), which made traditional soaps hardly practical.

The period that followed this reference date can be characterized by:

▪ steadily increasing interest in the knowledge of colloidal competencies and structural diversification of the range of nonionic soaps;

▪ major interest for the aliphatic series against the aromatic;

▪ dissemination of research results preferentially in the patent literature of Europe (Germany) and U.S.A.

**Figure 2:**
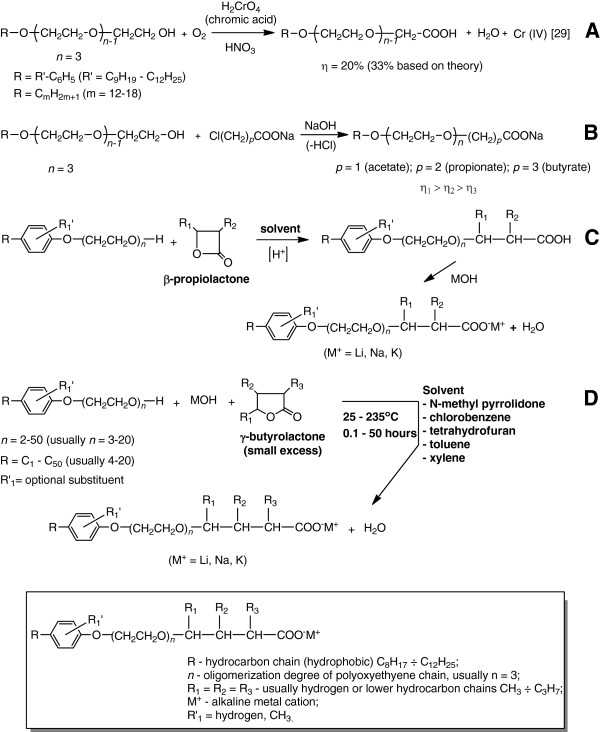
Procedures for obtaining nonionic soaps.

Currently the following procedures for obtaining nonionic soaps are frequently recorded (Figure 2):

1. Oxidation of linear polyethoxylated (n = 3-20) higher alcohols (C_12_H_25_-C_18_H_37_) and of polyethoxylated nonylphenols (n = 3-20): with chromic and nitric acid [16]; in the presence of titanium dioxide in basic medium (additional conditions not specified) [17], and catalytic oxidation with platinum, palladium and/or other metals-based catalysts, respectively [18], when together with the corresponding nonionic soaps, the presence of free higher alcohols due to the scission of etheric “bridges” is confirmed (Figure 2A);

2. Reaction of linear polyethoxylated (n = 3-20) higher alcohols (C_12_H_25_-C_18_H_37_) in basic medium with anhydrous alkaline or alkaline earth salts of monochloroacetic/ monochloropropionic acid at molar ratios in the range of 1/0,7-1/1,25, at 20-140°C at atmospheric pressure [18]. According to the literature data [19], the strongest effect on the ionization of the carboxyl group is exerted by the chlorine atom in the α position to the group. The influence decreases with distance, being weaker in the β position than in α and even weaker in the γ position, and therefore η_1_ > η_2_ > η_3_ (Figure 2B). According to the electronic theory, the increased acidity of halogenated acids, when compared with the corresponding non-halogenated acids, is due to the electron-attracting inductive effect of the halogen atoms (−I effect), the more stronger as it is located closer in the chain to the carboxyl group.

3. Reaction of polyethoxylated (n = 2-50 ethylene oxide units) nonylphenols with an alkaline hydroxide at 100-120°C with the intermediate formation of the corresponding phenoxide, which subsequently with γ-butyrolactone at 100-110°C forms with yields exceeding 85% a range of nonionic surface-active carboxybutyric compounds [20] (Figure 2D). Similarly, using β-propiolactone (Figure 2C) nonionic carboxypropionic soaps can be synthesized.

Between 1966–1974 Yoshiro Abe and collaborators [21-24] at Keio University (Japan) report the processing of surface-active structures derived from β-substituted propionic acids by employing the cyanoethylation reaction. In the same field of activity C. Jianu [25-30] communicates the obtaining of nonionic soaps and “mimetic lipids” also employing the cyanoethylation reaction of the aliphatic and/or aromatic hydroxyl functional group.

**Figure 3:**
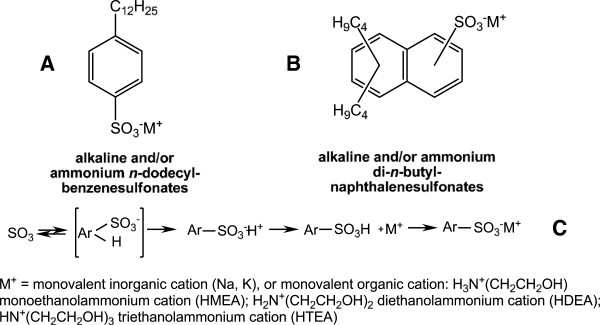
Surface-active alkaline and/or ammonium alkylarylsulfonates (A, B) and the diagram of the aromatic sulfonation process (C).

**Figure 4:**
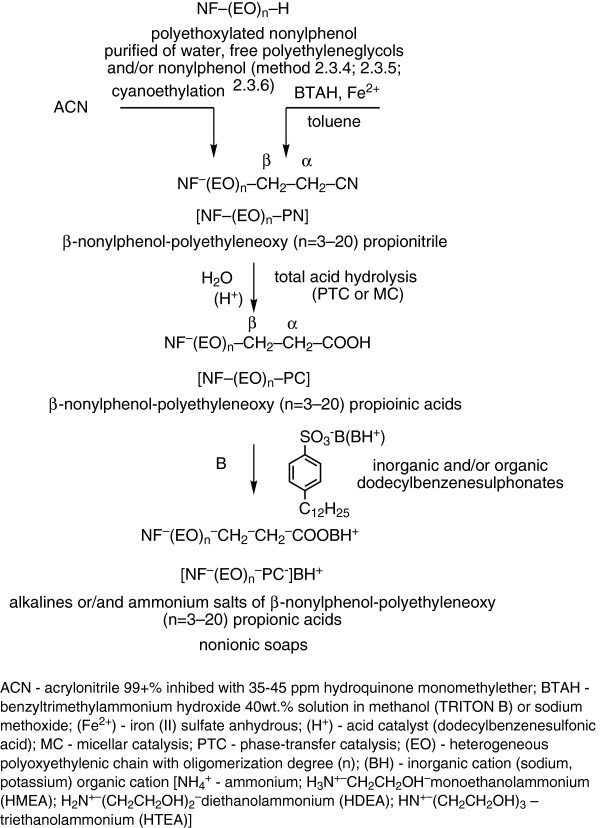
The processing scheme of alkaline or/and ammonium salts of β-nonylphenoxy polyethyleneoxy (n=3-20) propionic acids, as nonionic soaps.

Alkaline and/or ammonium alkylarylsulfonates (n-dodecylbenzenesulfonates) (Figure 3A), anionic surface-active compounds, are traditional components of the active substance in many washing/food sanitizing receipts. Sulfonation of aromatic rings (in benzene and/or naphthalene) is widely used in the technological practice in the processing of wetting agents, emulsifiers, demulsifiers (Figure 3A and 3B). It is a reversible process of unimolecular electrophilic substitution (SE1), in which the actual sulfonation agent is SO_3_ (Figure 3C). Dodecylbenzenesulfonic acid (DBSH) in technical products from the technological practice is present in proportion of over 98% together with ca. 2% residual sulfuric acid. In this study both components were acid catalysts in the exhaustive hydrolysis of β-nonylphenoxy (NF) polyethyleneoxy (n = 3-20) propionitriles [NF(EO)_n_-PN)] to β-nonylphenoxy (NF) polyethyleneoxy (n = 3-20) propionic acids [NF(EO)_n_-PC)] (Figure 4).

In this paper a sequence of classical reactions is adopted for the first time as the operating strategy, constructively adapted to obtain a new product range of nonionic soaps based on alkaline and/or ethanolammonium β-nonylphenolpolyethyleneoxy (n = 3-20) propionates. In this scheme, cyanoethylation of the hydroxyl functional group substitutes the oxidation reaction and/or the reaction with sodium monochloroacetate with the following major benefits: eliminates the byproducts difficult to monitor as diversity and share of the oxidation products (radical reactions); eliminates the increased corrosion of industrial installations due to hydrochloric acid (reaction with sodium monochloroacetate). In the proposed draft version the accidental formation of oligomers of the acrylic monomer under alkaline catalysis is inhibited, through the action of FeSO_4_ (Fe^2+^) associated with the other working parameters, and practically quantitative yields of nucleophilic addition are ensured.

This work reports for the first time the importance of certain impurities (water, polyethyleneglycols, free nonylphenol) in the technical products and studies after prior selective purification their qualitative and quantitative influence on the processing yields by controlled subsequent additivation.

In the conducted study for the first time is reported and argued the different development of the cyanoethylation of free nonylphenol compared to that of polyethoxylated nonylphenols (n = 3-20), highlighting the favorable role of polyoxyethylene chains grafted at oligomerization degrees n ≥ 3. In the same context is emphasized the decisive role of phase-transfer catalysts in the first case.

In the conducted study is accepted as operating premise and confirmed for the first time the favorable effect of dodecylbenzenesulphonic acid in the exhaustive acid catalysis of β-substituted propionates, but also in the final structuring of a flexible binary nonionic-ionic colloidal system with real possibilities of implementation in food sanitation systems.

## Results and discussions

A continuation of the colloidal optimization preoccupations and diversification of nonionic soaps as such and/or in combination [26,30], this work is aiming at the controlled simultaneous obtaining of surface-active mixtures based on alkaline and ammonium β-nonylphenoxy (NF) polyethyleneoxy (n = 3-20) propionates/dodecylbenzenesulfonates by following the diagram of operations in Figure 4.

Knowing the factors that influence the evolution of the processes shown in the block diagram of operations (Figure 4) is of great importance in the analysis of basic mechanisms of reactions in the “boundary layer”, for the intensification of mass and/or energy transfer coefficients by increasing the contact surfaces in heterogeneous processes (hydrolysis), and reducing the thickness of interphase separation interfaces etc., respectively.

Knowing the main stage parameters that may affect the processing yields and/or composition of the final reaction mixture, the mutual dependence was followed through the gradual, successive change of one of them and keeping the other constant in order to maximize the share of the main reaction product, β-substituted propionitriles and/or β-nonylphenolpolyethyleneoxy (n = 3-20) propionic acids, and to minimize the byproducts (mainly oligomers and/or polymers of the acrylonitrile monomer), respectively.

The resulting experimental database enabled the mathematical processing and the determination of the nature of the dependence equations (linear, logarithmic, exponential or polynomial) with the maximum correlation coefficient.

**Figure 5:**
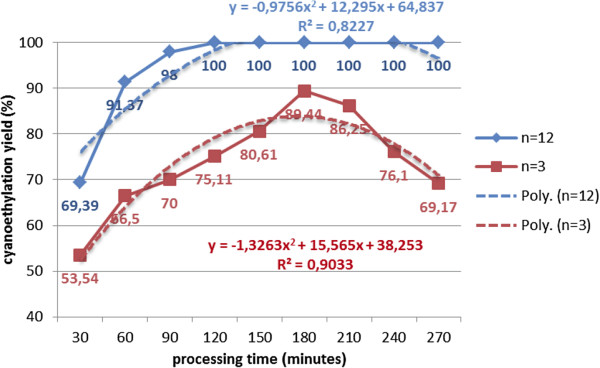
Dependence of the cyanoethylation yield of polyethoxylated nonylphenols, expressed as the ratio NF(EO)_n=3,12_PN (%) / acrylic oligomers (%), on the processing time, solvent toluene, temperature 30°C, molar ratio acrylonitrile/NF(EO)nH 1.1/1, concentration of basic catalyst [CH_3_O^-^Na^+^] 5×10^-3^ mol/L, without polymerization inhibitor.

**Figure 6:**
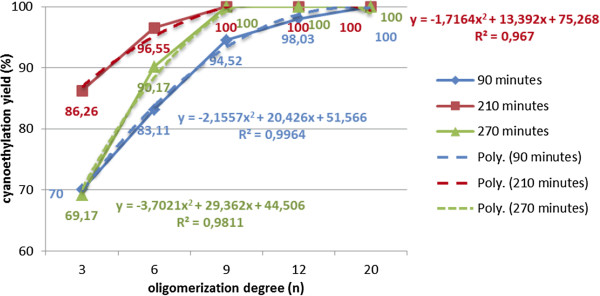
Dependence of the cyanoethylation yield of polyethoxylated nonylphenols, expressed as the ratio NF(EO)_n=3,12_PN (%) / acrylic oligomers (%), on the oligomerization degree (n) for 90, 210, 270 minutes processing time, solvent toluene, temperature 30°C, molar ratio acrylonitrile/NF(EO)nH 1.1/1, concentration of basic catalyst [CH_3_O^-^Na^+^] 5×10^-3^ mol/L, without polymerization inhibitor.

**Figure 7:**
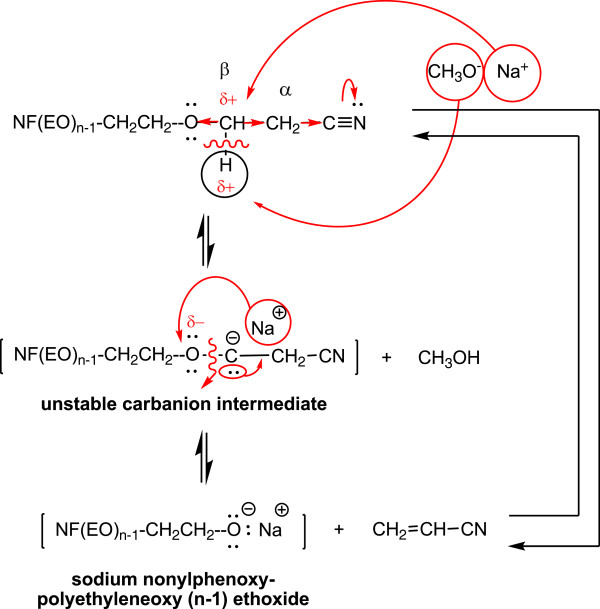
The probable splitting scheme of the newly formed “ether bridge” in β-nonylphenolpolyethyleneoxy (n=3-20) propionitriles under prolonged contact (over 180 minutes) with the basic catalyst [CH_3_O^-^Na^+^].

In the process of cyanoethylation of polyethoxylated nonylphenols, the reaction time favors the formation of β-substituted propionitriles [R(EO)nPN] up to 180 minutes, and the oligomerization of the acrylic monomer throughout the process (Figure 5,6). After this period, the cyanoethylation yields decrease due to their prolonged contact with the basic medium, suggesting the reversible character at prolonged contact between the reactants (Figure 7). Its maximum value is achieved at lower reaction times as the oligomerization degree (n) of the polyoxyethylene chain increases, which may suggest the involvement of the polyoxyethylene chain in the process (Figure 6).

**Figure 8:**
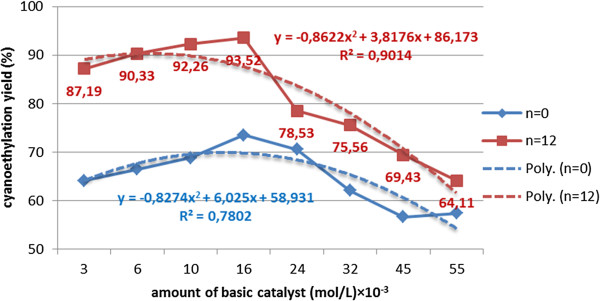
Dependence of the cyanoethylation yield of polyethoxylated nonylphenols (n=0;12), (nitrile/acrylic oligomers %), on the amount of catalyst, solvent toluene, processing time 60 minutes, temperature 30°C, molar ratio acrylonitrile/NF(EO)_n_H 1.1/1, without oligomerization inhibitor.

**Figure 9:**
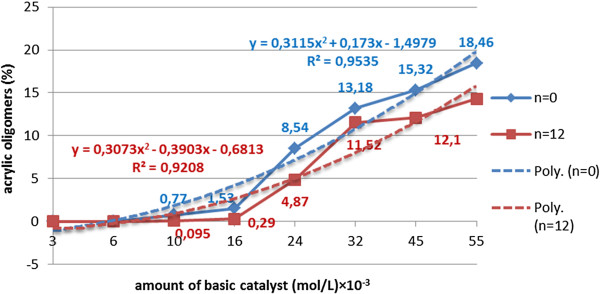
Dependence of the amount of acrylic oligomers on the amount of basic catalyst (CH_3_O^-^Na^+^) (mol/L)×10^-3^ at cyanoethylation of polyethoxylated nonylphenols (n=0;12) solvent toluene, processing time 60 minutes, temperature 30°C, molar ratio acrylonitrile/NF(EO)_n_H 1.1/1, without oligomerization inhibitor.

**Figure 10:**
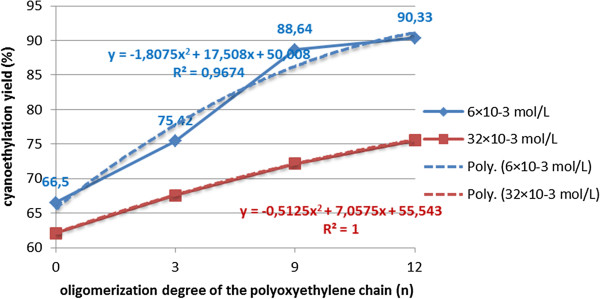
Dependence of the cyanoethylation yield of polyethoxylated nonylphenols (n=0-12) on the oligomerization degree of the polyoxyethylene chain (n) for the same amount of basic catalyst (CH_3_O^-^Na^+^) ×10^-3^ mol/L at two extreme values (6×10^-3^ mol/L; 32×10^-3^ mol/L), solvent toluene, processing time 60 minute, temperature 30°C, molar ratio acrylonitrile/NF(EO)_n_H 1.1/1, without oligomerization inhibitor.

Increasing the amount of catalyst above the optimal value (4-5·10^-3^ mol/L) increases the alkalinity of the environment and favors the oligomerization reactions of the acrylic monomer. In the concentration range of 15-50·10^-3^ mol/L the content of [R(EO)_n_PN] decreases, in parallel with the rapid growth of the oligomers content. In the series of polyoxyethylene chain homologues, the maximum cyanoethylation yield is reached at limited values of catalyst concentration. At the same concentration of catalyst the dimensional increase of the polyoxyethylene chain determines a sensible increase of the nucleophilic addition yields, but also a reduction in the amount of oligomers formed, probably due to the solvation of the acrylic monomer in the polyether chain (Figures 8, 9, 10).

**Figure 11:**
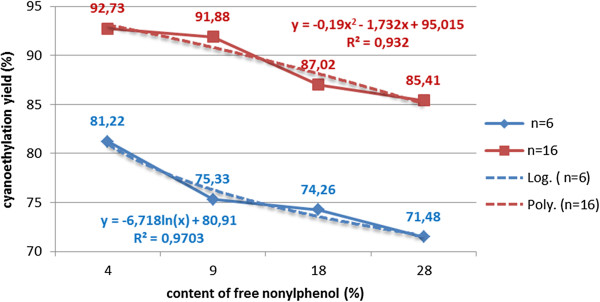
Dependence of the cyanoethylation yield of polyethoxylated nonylphenols (n=6;16), on the content of free nonylphenol, molar ratio acrylonitrile/NF(EO)_n_H 1.1/1, processing time 60 minutes, solvent toluene, temperature 30°C, catalyst 5x10^-3^ mol/L, without oligomerization inhibitor.

The initial presence of free nonylphenol in varying amounts (5-40%) negatively influences the cyanoethylation yields through the equivalent monomer consumption and the lower cyanoethylation rate [the nucleophilicity of the alkaline nonylphenoxide decreases due to the (−Is) and (+Es) effects, respectively, of opposite sign]. The phenomenon can be explained on the one hand through the “steric hindrance” effect due to the hydrocarbon chain, and through the lower nucleophilic activity of the alkaline nonylphenoxide, compared to that of the polyethoxylated nonylphenols (Figure 11).

**Figure 12:**
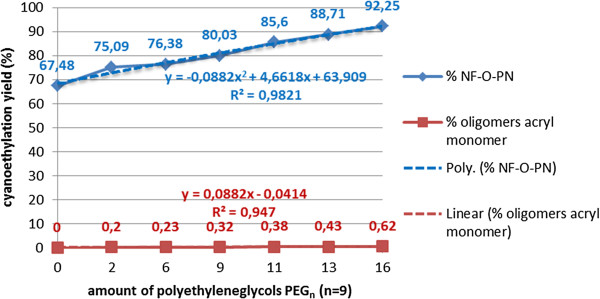
Dependence of the cyanoethylation yield of free nonylphenol, on the amount of polyethyleneglycols, PEG_n_ (n=9), molar ratio acrylonitrile/free nonylphenol 1.1/1, processing time 60 minutes, solvent toluene, catalyst concentration 5x10^-3^ mol/L, temperature 25°C, without oligomerization inhibitor.

The presence of polyethyleneglycols in varying amounts (1-15%) in the processing mixture (Figure 12) of polyethoxylated nonylphenols, influences the cyanoethylation yields in two opposite directions:

▪ high consumption of monomer (1–2 moles/mol PEG) with formation of polyethyleneglycol (n = 3-30) dipropionitriles (dicyanoethylated polyethyleneglycols);

▪ participation of dicyanoethylated polyethyleneglycols in the cyanoethylation process, as mono- or diprotected oligoglymes (PTC) (activation of the alkaline nonylphenoxides through the coordination of the alkaline cation). The favorable contribution to the cyanoethylation yields depends on the average degree of polyethoxylation of nonylphenol (n), since the distribution of polyoxyethylene chain homologues is larger, as the nonylphenol is more highly polyethoxylated, and the distribution of PEG homologues in the (n = 3-30) range is wider than that of the homologues in NF-(EO)_n_-H (n = 3-20), for the same average ethylene oxide content.

In the processing conditions mentioned were found increased amounts of:

▪ oligomers in the final reaction mixture, because alkaline polyethyleneglycolates, as bidentate nucleophilic agents, have higher alkalinity than alkaline nonylphenoxides (Figure 12);

▪ β-nonylphenoxy propionitriles, proportional to the amount of polyethyleneglycols, due to the favorable effect of phase-transfer catalysis (Figure 12)

**Figure 13:**
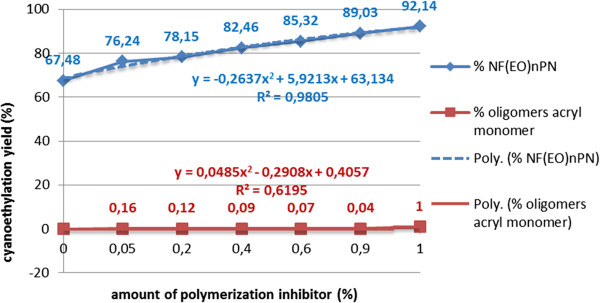
Dependence of the cyanoethylation yield of free nonylphenol, on the content of polymerization inhibitor (anhydrous FeSO_4_), molar ratio acrylonitrile/free nonylphenol 1.1/1, temperature 30°C, processing time 2 h, solvent toluene, catalyst concentration 5x10^-3^ mol/L.

In the conducted study the formation of oligomers of acrylonitrile in basic medium was avoided through the introduction of anhydrous ferrous sulfate (FeSO_4_) (Figure 13) as inhibitor of the oligomerization/polymerization, respectively, of the acrylic monomer. For the addition of 1% inhibitor, the yield of the cyanoethylation of free nonylphenol increases by more than 10%, without the formation of oligomers. Similar results are also obtained in the homologous series of polyethoxylated nonylphenols NF(EO)_n_H (n = 3-20).

The polyoxyethylene chain, with its specific conformation [31], intervenes in the cyanoethylation process in nonpolar reaction media through the activation of the nucleophile, so that the processing yields for polyethoxylated nonylphenols, with the same polyoxyethylene chain in the homologous series (n = 3-20), increase.

As confirmed experimentally the hypothesis of active participation of the polyoxyethylene chain in the cyanoethylation process allowed the approximation of the coordination numbers of the alkali cations with the phase-transfer catalyst (GLYME-n-PN) in the class of “homogeneous” dicyanoethylated polyethyleneglycols (n = 3, 6, 9, 12, 16, 20). (The notion of phase transfer is referring to the movement through the interface of the alkaline cation sequestered in the “reverse” micelle/medium system).

From the analysis of experimental data it can be stated that the cyanoethylation of free (NFOH) and/or polyethoxylated [NF(EO)_n_H] nonylphenol proceeds by a complex mechanism, in which the rate-determining step is the actual nucleophilic addition. Its precursive and subsequent steps are protolytic equilibria (acid–base), in which the nucleophilic agent characteristic to the hydroxyl substrate is formed, or the carbanionic structure resulting from the addition is stabilized, respectively.

Since in the formation of the activated complex are involved a polarized (the acrylic monomer) and an ionic (alkaline nonylphenoxide) molecular species, the reaction is strongly influenced by solvation effects (coordination), due to both the polarity of the medium and the nature of the alkaline cation. The increase of the ionic character of the nucleophile-alkaline cation bond favors the coordination effects specific to the conformation of polyoxyethylene chains (n = 3-30), therefore comparable with similar diprotected chains (dicyanoethylated polyethyleneglycols). Simultaneously with the reaction rate increases the monomer consumption. Determination of the partial reaction orders with respect to glymes allowed the indirect estimation of the size of the “elementary cell” of coordination of the alkaline cation to the value of 8–9.

Determination of the partial reaction orders with respect to glymes allowed the indirect estimation of the size of the “elementary cell” of coordination of the alkaline cation to the value of 8–9 [26,32-34]. The statements expressed and mechanisms of action formulated represent our own interpretations of the experimental data recorded. They are supported by the results, comments, discussions and literature on the currently widely accepted geometry and spatial conformation of the acyclic polyoxyethylene chains with various degrees of oligomerization (n) [35-39].

A reversible reaction, cyanoethylation is determined by temperature, duration, excess reactant (monomer) and the addition products. Secondary products existent in the raw materials (free nonylphenols, polyethyleneglycols, traces of water) and the oligomers of acrylonitrile in the reaction product, negatively affect the yields of the nucleophilic addition through the monomer consumption and the subsequent purification difficulties. That is why the presence of oligomerization inhibitors (FeSO_4_) is necessary and recommended.

Compared to the previous preliminary research [26,30], the exhaustive acid hydrolysis of β-nonylphenoxy-polyethyleneoxy (n = 3-20) propionitriles was conducted in the presence of dodecylbenzenesulfonic acid (DBSH), a strong organic acid (comparable with mineral acids), miscible with water. During the processing the scission of the sulfonic functional group in DBSH in the presence of traces of H_2_SO_4_ (1-2%) in the acid catalyst was not observed in the temperature range 100-160°C.

The heterogeneous nature of the acid hydrolysis systems affects the contact between the reactants (β-nonylphenoxy-polyethyleneoxy-propionitriles, water and catalyst), therefore the prior indicative evaluation of the HLB balance (hydrophilic/hydrophobic balance) was imposed, and that of the water solubility of β-substituted propionitriles and propionic acids as reaction products, respectively (Table 1).

**Table 1:** Reference physico-chemical parameters for the colloidal behavior of β-nonylphenoxy (NF) polyethyleneoxy (n=3, 6, 9, 12, 16, 20) propionic acids

**No.**	**Average oligomerization degree of ethylene oxide (n)**	**Number of carbon atoms (N)**	**Average content of ethylene oxide (%EO)**	**HLB index (GRIFFIN) HLB =**$\frac{\%EO}{5}$	**Solubility in aqueous medium**^**1)**^$\frac{\%EO}{N}$
1	3	24	31,13	6,226	1,297
2	6	30	47,48	9,49	1,582
3	9	36	57,56	11,51	1,598
4	12	42	64,39	12,88	1,533
5	16	50	70,68	14,14	1,413
6	20	58	75,58	15,02	1,294

**Legend**: ^1)^ Betweeen 0.5-1.5 soluble within certain limits; above 1.5 higly soluble [32,33].

From the analysis of data presented in Table 1 the following aspects can be observed:

▪ the HLB balance increases with the hydrophilicity (the polyethoxylation degree of nonylphenol) of the β-substituted propionitriles;

▪ their solubility evaluated by the value of the %EO/N ratio [40,41] and verified practically by determining the water number (Karabinos method) is high.

**Figure 14:**
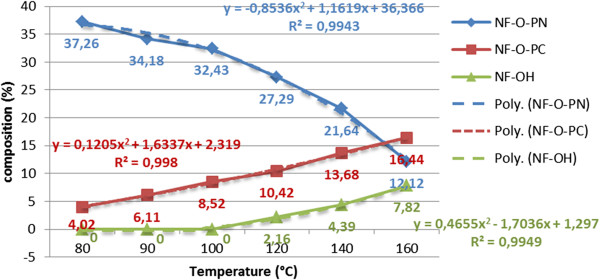
Dependence of the composition and yields of total acid hydrolysis (acid catalyst DBSH) of β-nonylphenoxypropionitrile on temperature in the range 80-160°C for a molar ratio DBSH/NF-O-PN 1/1, without phase-transfer catalyst (PTC), processing time 60 minutes, molar ratio water/NF-O-PN 2/1.

In order to assess these influences, initially the acid hydrolysis was studied on β-nonylphenoxy (NF) (n = 0) propionitrile (NF-O-PN), immiscible with water. In this respect, the temperature dependence of the composition of the processing mixture was followed within the range 80-160°C (Figure 14), initially without the influence (presence) of the phase-transfer catalyst. From the comparative analysis of the experimental data it is noted that the yield of nonylphenoxypropionic acid (NF-O-PC) increases throughout the specified temperature range. The content of nonylphenoxypropionitrile (NF-O-PN) decreases practically, while the amount of free nonylphenol increases predominantly in the range 120-160°C due to the splitting in strong acidic medium of the “ether bridge” formed in the cyanoethylation step.

Under the specified conditions the evolutions of the proportions of NF-O-PN and NF-O-PC, respectively, for a molar ratio DBSH/NF-O-PN 1/1, without PTC suggests a downward trend for the propionitrile and an upward one for the content of propionic acid. Simultaneously with the increase of temperature in the range 80-160°C this trend is maintained, but the increase of the free nonylphenol content is also noted. This confirms the reversible character of the hydrolysis process due to the scission in strongly acidic medium at temperatures above 120°C of the “ether bridge” in NF-O-PN.

**Figure 15:**
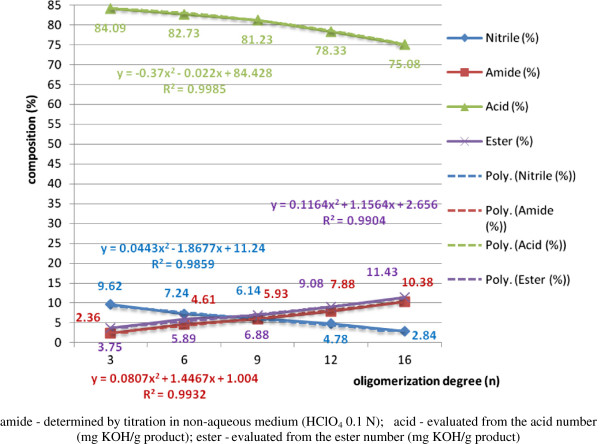
Dependence of the composition of the mixture of total acid hydrolysis of β-nonylphenoxypolyethyleneoxy (n=3-16) propionitriles contaminated with 2% PEG_9_ on the degree oligomerization of the polyoxyethylene chain (n), molar ratio dodecylbenzenesulphonic acid/β-nonylphenoxy polyethyleneoxy (n=3-16) propionitrile [NF(EO)_n_PN] 0.5/1, processing time 60 minute, temperature 80°C, molar ratio [water/NF(EO)_n_PN] 2/1, without phase-transfer catalyst.

**Figure 16:**
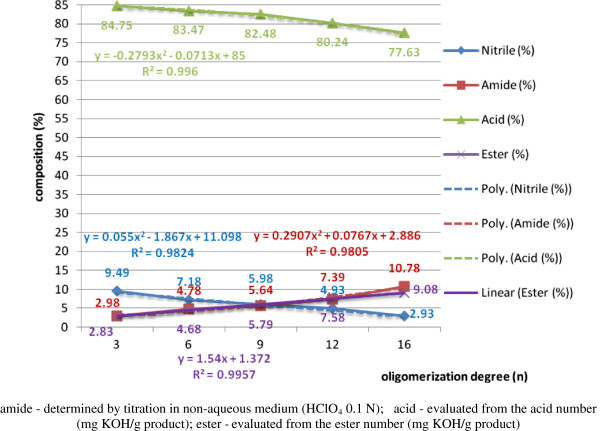
Dependence of the composition of the mixture of total acid hydrolysis of β-nonylphenoxypolyethyleneoxy (n=3-16) propionitriles contaminated with 4% NFOH on the degree oligomerization of the polyoxyethylene chain (n), molar ratio dodecylbenzenesulphonic acid/β-nonylphenoxy polyethyleneoxy (n=3-16) propionitrile [NF(EO)_n_PN] 0.5/1, processing time 60 minute, temperature 80°C, molar ratio [water/NF(EO)_n_PN] 2/1.

The dependence of the composition of the processing mixture and of conversion yields, respectively, on the operating parameters and/or structural factors for the heterogeneous homologous series of polyethoxylated nonylphenols NF(EO)_n_-H (n = 3, 6, 9, 12, 16, 20) was performed initially on technical products purified of water, free nonylphenol and/or polyethyleneglycols through repeated liquid/liquid extractions in appropriate solvent systems. In order to determine the influence of these secondary products on the processing yields, in this work experiments were performed with controlled addition (2%) of polyethyleneglycols (PEG_6_) with an oligomerization degree (n = 6) (Figure 15) and free nonylphenol (NF-OH) (4%), respectively (Figure 16).

The controlled sequential additivation of these secondary products in the exhaustive acid hydrolysis step of β-nonylphenoxypropionitrile (NF-O-PN) affects overall the composition and processing yields by lowering the yields of β-nonylphenoxypropionic acid (NF-O-PC) at the expense of the esters NF-O-PE formed through the derivatization of the hydroxyl terminals in PEG and NF-OH.

If nonylphenoxypropionitrile is hydrolyzed with initial addition (cyanoethylation step) of polyethyleneglycols, the post-hydrolysis final mixture is even more complex due to the esters also formed by the exhaustive acid hydrolysis of nitrile functional groups in dicyanoethylated polyethyleneglycols and cyanoethylated nonylphenol, respectively.

Water was removed from the technical processes before processing by heating under effective stirring (without foaming) at 80-90°C under vacuum (10^-2^-10^-3^ mm Hg). The content of free nonylphenol in the initial technical polyethoxylated product varies inversely with the oligomerization degree of ethylene oxide grafted on the hydroxyl functional group. From Figure 15 it is observed that together with the increase of the oligomerization degree in the homologous series n = 3 → 16, the proportion of NF(EO)_n_PN and NF(EO)_n_PC, β-nonylphenoxypolyethyleneoxy (n = 3-20) propionic acids, decreases continuously, while the proportion of β-nonylphenoxypolyethyleneoxy (n = 3-20) propionic acid esters, NF(EO)_n_PE, increase continuously in the same order. In the case of β-nonylphenoxypolyethyleneoxy (n = 3-20) propionamides [NF(EO)_n_PD], their concentration increases initially (60 minutes), after which reaches a maximum and decreases continuously until the disappearance from the processing medium, initially due to the partial acid hydrolysis of NF(EO)_n_PN to NF(EO)_n_PD, and later due to the partial acid hydrolysis of NF(EO)_n_PD to NF(EO)_n_PC. The relatively low amount [2-12% NF(EO)_n_PE] is due to the esterification under the specified conditions of NF(EO)_n_PC with NFOH present through the controlled additivation, and not due to the scission of the “ether bridge” under the pH and temperature conditions of the processing. In comparison with the processing attempts with polyethoxylated nonylphenols (n = 3-20) not purified from PEG and NFOH, the same processing yields are lower for NF(EO)_n_PN and NF(EO)_n_PD, as the acrylonitrile monomer is partially consumed in the derivatization of polyethyleneglycols (cyanoethylation) at the two hydroxyl terminals and the cyanoethylation of free nonylphenol, respectively. The final processing mixtures are more complex also in their colloidal behavior, together with the change of the hydrophilic/hydrophobic balance of the initial binary system.

In this work an increasing percental molar ratio of the acid catalyst (DBSH) was used (0.1/1-4/1) in relation to β-nonylphenoxypolyethyleneoxy (n = 3-20) propionitrile to ensure in the end, after stoichiometric neutralization with inorganic or organic bases, respectively, the controlled proportion of the anionic surface-active component in the proposed binary colloidal system.

Depending on the working conditions and those of the processing of the reaction products, two classes of compounds were obtained: the free acids or their salts [NF(EO)_n_PC, (soaps)] with surface-active properties.

**Figure 17:**
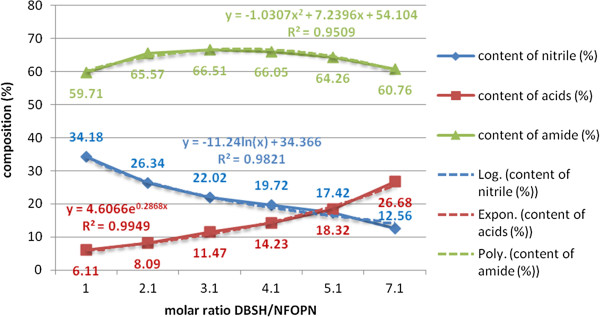
Dependence of the yields of total acid hydrolysis (acid catalyst DBSH) of β-nonylphenoxypropionitrile on the molar ratio DBSH/NF-O-PN, processing time 60 minutes, temperature 90°C, molar ratio water/NF-O-PN 2/1 without PTC_2_/PTC_3_ system.

**Figure 18:**
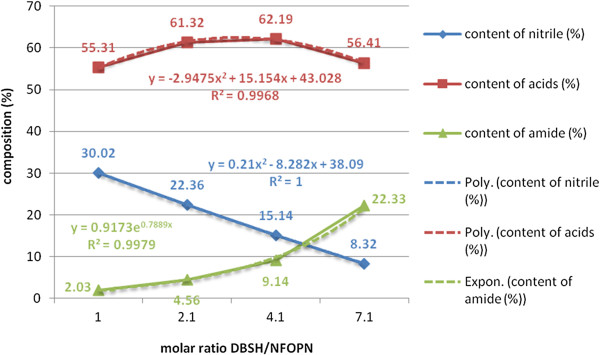
Dependence of the yields of total acid hydrolysis (acid catalyst DBSH) of β-nonylphenoxypropionitrile on the molar ratio DBSH/NF-O-PN, processing time 60 minutes, temperature 90°C, molar ratio water/NF-O-PN 2/1 with PTC_2_/PTC_3_ system.

The evolution of the total hydrolysis yields was negatively affected by the heterogeneity of the processing environment (biphasic system), but favorably by the phenomena of micellar catalysis and/or in emulsion and of phase-transfer catalysis (PTC), respectively (Figures 17, 18).

**Figure 19:**
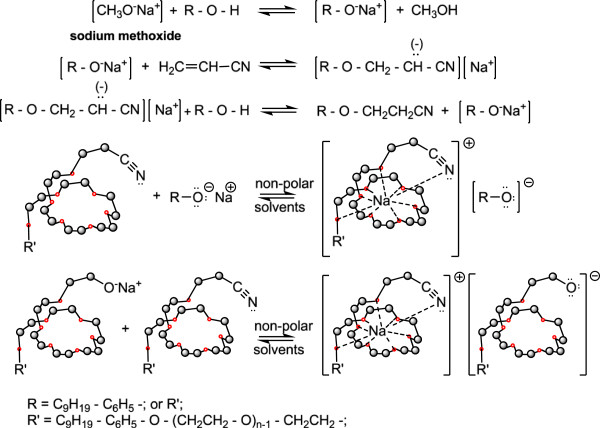
Scheme of the principle of coordination in non-polar solvents of alkali cations in the cavity of the polyoxyethylene chain with 8–9 oxygen atoms of β-nonylphenoxy-polyethyleneoxy-propionitriles.

**Figure 20:**
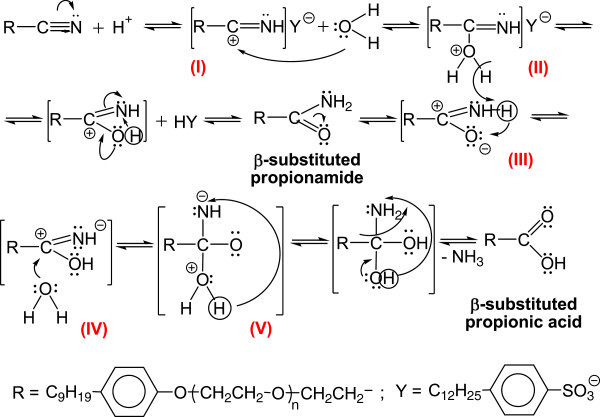
Probable mechanism for the exhaustive acid hydrolysis of β-nonylphenoxypolyethyleneoxy (n=3-20) propionitriles (β-substituted propionamide; β-substituted propionic acid).

The idea of checking the influence of the phase-transfer catalysts on the exhaustive acid hydrolysis emerged after finding that in the previous cyanoethylation process, the yields increase simultaneously with the oligomerization degree of the polyoxyethylene chain in the homologous series n = 3, 6, 9, 12, 16, 20 (Figure 19). This observation was explained by its active participation in the activation of the reaction intermediates with cationic structure I-V in Figure 20 by the “sandwich” and/or helical conformation of the polyoxyethylene chains [14], depending on the size of the oligomerization degree. The previously formulated hypothesis was confirmed by studying the evolution of the yields of the exhaustive acid hydrolysis of β-nonylphenoxy (n = 0) propionitrile [NF-O-PN] with and without the addition of PTC_1-4_. The presence of PTC in the hydrolysis medium increased the reaction yields by approx. 4% (Figures 17, 18).

In all cases, above 80°C the content of β-nonylphenoxy (NF) polyethyleneoxy (n = 3, 6, 9, 16, 20) propionitrile drops sharply regardless of the acid catalyst used, and the continuous increase of the presence of propionic esters is observed. Increasing the processing time favors the total acid hydrolysis. Above 80°C (particularly in the version of hydrochloric acid catalysis), after ca. 60 minutes β-nonylphenoxy (n = 0) propionitrile could not be found in the reaction mixture, and after 90 minutes neither the intermediately formed propionamide.

**Figure 21:**
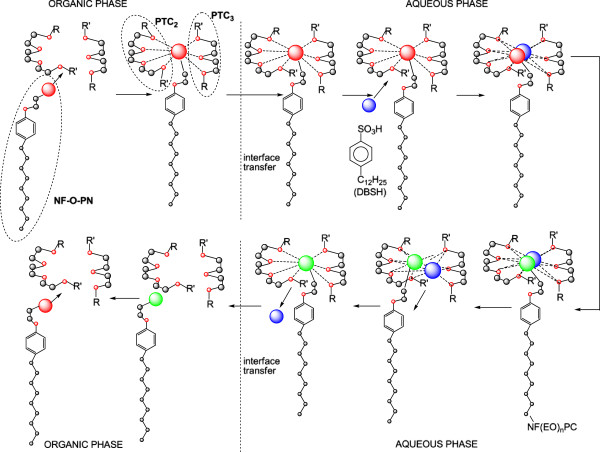
**Graphical representation of the steps of exhaustive acid hydrolysis (PTC_2_/PTC_3_), “sandwich model” under phase-transfer catalysis (PTC) of β-nonylphenoxypolyethyleneoxy (n=3-20) propionitriles [NF(EO)_n_PN)] to β-nonylphenoxypolyethyleneoxy (n=3-20) propionic acids [NF(EO)_n_PC)] Mathematical correlation of the experimental data enabled the generalization of a particular casuistry through mathematical relationships (linear, exponential, logarithmic and/or polynomial) correlation coefficients R^2^ in the range 0.9-1.** Thus are confirmed the reproducibility of the values recorded and the accuracy of the interpretations made.

In Figure 21 is presented in a schematized manner the probable coordination (sequestration) mode of the intermediates with cationic character (I-V) in Figure 20, in the “sandwich” model for the spatial conformation of PTC_2_ and PTC_3_ in order to be transferred through the interface separating the two phases (media) (organic and aqueous). The unitary operation is favored by the concentration potential difference between them, but also by the miscibility of the systems formed in both phases. In the aqueous phase the acid catalyst (DBSH) also joins in the sequestration space, favoring the exhaustive acid hydrolysis (Figure 20) of β-nonylphenoxypolyethyleneoxy (n = 0-20) propionitriles [NF-O-PN; NF(EO)_n_PN] to β-nonylphenoxypolyethyleneoxy (n = 0-20) propionic acids [NF-O-PC; NF(EO)_n_PC]. Simultaneously with the movement in the aqueous phase the recovery of the acid catalyst occurs, as well as its separation from the β-substituted propionic acid formed, the transfer through the separation interface and the preparation of the system for a new cycle of assisted exhaustive acid hydrolysis.

**Figure 22:**
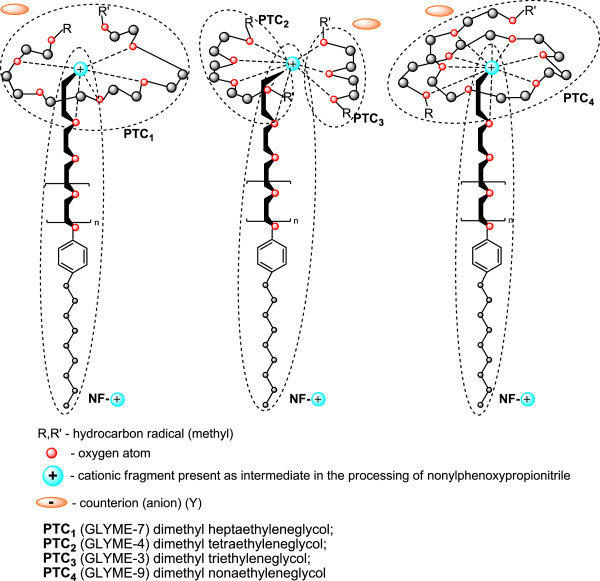
**Conformational structures of the phase-transfer catalysts (PTC_1-4_) used in the exhaustive acid hydrolysis of nonylphenoxypropionitriles [**[26]**].**

The experimental attempts conducted with PTC_1_ and PTC_4_, respectively, led to similar results, confirming that all coordination models, “cage”, “sandwich” (Figure 21) and “tunnel” (Figure 22), favor the exhaustive acid hydrolysis.

### Experimental

Materials:

▪ Heterogeneously polyethoxylated nonylphenols (n = 3-20) (technical products of S.C. Romtensid S.A.) purified of water, polyethyleneglycols, free nonylphenol [25-30] (Table 2). The content of free nonylphenol (NFOH) determined was inversely proportional to the oligomerization degree [e.g., for n = 3 the content of (NFOH) was 23.29%; for n = 6, 2.14%, for n = 9, 1.72%, for n = 12, 1.54%, for n = 16, 1.26%, and for n = 20. 0.85%, respectively] [38,39].

▪ β-nonylphenoxy (NF) polyethyleneoxy (n = 3-20) propionitriles (technical products), 97-98% purity [26];

▪ β-nonylphenoxy (NF) polyethyleneoxy (n = 3-20) propionic acids (technical products), 97-98% purity [26];

▪ Alkaline and/or ammonium salts of β-nonylphenoxy (NF) polyethyleneoxy (n = 3-20) propionic acids (technical products, 98% purity) [26];

▪ Phase-transfer catalysts (PTC) (“homogeneous” mono- and/or di-derivatized polyoxyethylene chains) [26] (Figure 22).

**Table 2:** The main physico-chemical characteristics of heterogeneously polyethoxylated (n=3, 6, 9, 12, 18) nonylphenols (NF), purified of water, polyethyleneglycols, free nonylphenol

**No.**	**Average oligomerization degree (n)**		**Molecular weight (M)**	**Content of ethylene oxide (%)**		**Hydroxyl number [mg KOH/g heterogeneous polyoxyethylene (PEO) chain monoderivatized NF]**		**Purity**^**1)**^**(%)**
	**Determined**	**Calculated**		**Determined**	**Calculated**	**Determined**	**Calculated**	
1	2.99	3	352	37.41	37.50	158.69	159.09	99.75
2	5.99	6	484	54.43	54.54	115.47	115.70	99.80
3	8.96	9	616	64.01	64.29	90.52	90.91	99.57
4	11.96	12	748	70.36	70.59	74.63	74.87	99.68
5	17.95	18	1012	78.05	78.26	55.19	55.34	99.73

**Legend**: ^1)^ weighted average value reported simultaneously to the content of ethylene oxide and the hydroxyl number.

Reagents (Sigma-Aldrich, Merck) of analytical purity.

## Methods

### Preparation of β-nonylphenoxy (NF) polyethyleneoxy (n = 3-20) propionitriles

In a thermostated processing vessel, fitted with mechanical stirring and condenser, under inert atmosphere 0.1 moles polyethoxylated nonylphenol, purified of water, polyethyleneglycols and/or free nonylphenol and 0.0025 moles basic catalyst CH_3_O^-^Na^+^ are introduced at a convenient temperature (30-35°C), 0.0025 moles (0.38 g) finely divided anhydrous FeSO_4_ are suspended and 5.83 g (0.11 moles) acrylonitrile are added cautiously under effective stirring, over 30–45 minutes, so that the temperature of the mixture does not exceed the prescribed value (if necessary the processing vessel is cooled in an ice-water bath). The mixture is maintained under similar conditions for 2 hours for perfecting the process, the basic catalyst is neutralized with ca. 4.8 mL solution CH_3_COOH 5% and the precipitated salts are filtered hot; the viscous residue, a fluid of slightly yellow color, is purified if necessary of acrylonitrile oligomers by elution at 40-60°C on a silica gel column. The main characteristics of the β-nonylphenoxy (NF) polyethyleneoxy propionitriles obtained are shown in Table 3. The cyanoethylation yields based on the introduced polyethoxylated nonylphenol are practically quantitative.

**Table 3:** The main physico-chemical characteristics of β-nonylphenoxy (NF) polyethyleneoxy (n=3, 6, 9, 12, 16, 20) propionitriles [NF(EO)_n_–PN)]

**No.**	**Average oligomerization degree (n)**		**Content of ethylene oxide (%)**		**Content of nitrogen (%)**		**Purity (%)**
	**Determined**	**Calculated**	Determined^*)^	**Calculated**	**Determined**	**Calculated**	Determined^**)^
1	2,99	3	37,23	37,50	3,95	3,98	99,27
2	5,98	6	54,27	54,54	2,87	2,89	99,40
3	9,03	9	63,84	64,28	2,25	2,27	99,32
4	12,02	12	70,38	70,59	1,87	1,88	99,71
5	16,12	16	75,98	76,46	1,53	1,52	99,43
6	20,34	20	79,86	80,24	1,27	1,28	99,62

**Legend**: ^*)^ cleavage with hydriodic acid (d = 1.7-1.9); ^**)^ weighted average value reported simultaneously to the determined content of ethylene oxide and nitrogen.

### Total acid hydrolysis of β-nonylphenoxy (NF) polyethyleneoxy (n = 3-20) propionitriles with dodecylbenzenesulfonic acid (DBSH) under phase-transfer catalysis conditions

In a thermostated processing vessel, fitted with mechanical stirring, condenser, and dropping funnel, 0.1 moles β-substituted propionitrile [NF(EO)_n_-PN) are introduced under efficient homogenization, 0.4 moles acid catalyst (DBSH), 2.77 moles (50 mL) water and 0.01 moles phase-transfer catalyst (PTC_1-4_) are added at 60-70°C (Figure 22). The reaction is perfected at 90-100°C, ca. 2 hours, then the mixture is neutralized with finely divided NaOH and water is removed under vacuum (10–20 mm Hg). The residue obtained, purified by repeated alcoholic extractions or elution on an ion-exchange column, is salified exhaustively with inorganic and/or organic bases and are determined the content of β-nonylphenoxy (NF) polyethyleneoxy propionic acids [NF(EO)_n_-PC] by determining the acid number, and β-nonylphenoxy (NF) polyethyleneoxy propionates by antagonistic titration with N-benzyl-N,N-dimethyl-N-alkylammonium chloride 0.01 M solution [benzalkonium chloride, CAS 8001-54-5], as well as that of inorganic and/or organic dodecylbenzenesulfonates, respectively. The yields based on the nitriles introduced in hydrolysis are between 98-99%. The main physico-chemical characteristics of β-nonylphenoxy (NF) polyethyleneoxy (n = 3, 6, 9, 12, 16, 20) propionic acids are shown in Table 4.

**Table 4:** The main physico-chemical characteristics of β-nonylphenoxy (NF) polyethyleneoxy (n=3, 6, 9, 12, 16, 20) propionic acids [NF(EO)_n_-PC)]

**No.**	**Average oligomerization degree (n)**	**Content of ethylene oxide (%)**		**Acid number (mg KOH/g product)**		**Purity (%)**
		Determined^*)^	**Calculated**	**Determined**	**Calculated**	Determined^**)^
1	3	30,84	31,13	132,08	132,08	99,08
2	6	47,16	47,48	100,04	100,72	99,33
3	9	57,09	57,56	80,73	81,39	99,19
4	12	64,05	64,39	67,93	68,29	99,47
5	16	70,92	70,37	55,14	55,98	99,36
6	20	73,99	74,81	47,82	47,60	99,28

**Legend**: ^*)^ cleavage with hydriodic acid (d = 1.7-1.9); ^**)^ weighted average value reported simultaneously to the determined content of ethylene oxide and nitrogen.

### Preparation of the system of alkaline and/or ammonium salts of β-nonylphenoxy polyethyleneoxy (n = 3-20) propionic acids, in mixtures with dodecylbenzenesulfonic acid (X^+^ = Na; K; HMEA; HDEA; HTEA)

In a thermostated processing vessel, fitted with mechanical stirring and dropping funnel, 0.1 moles mixture in a controlled proportion of the corresponding β-nonylphenoxy (NF) polyethyleneoxy (n = 3-20) propionic acid and dodecylbenzenesulfonic acid, respectively, is introduced, then the neutralizing agent is added cautiously at 60-70°C, under continuous effective stirring, in a convenient form (inorganic bases as 30% aqueous solutions in 10% excess, and organic bases as such in a 1/1 molar ratio). After ca. 1 hour, the pH of the mixture is adjusted to the 7.5-8.5 value, water is removed under vacuum (10–20 mm Hg), and the residue is purified by repeated alcoholic extractions. The purity (active substance content) is determined by the antagonistic titration method with cationic surfactant solution [benzalkonium chloride, CAS 8001-54-5], 0.01 M. Thereafter aqueous solutions with concentrations between 0.5-5 g/L were used to evaluate the main surface-active properties.

### Determination of the water content of polyethoxylated nonylphenol (n = 3-20), technical product

10–15 g polyethoxylated nonylphenol (n = 3-20), technical product, weighed with analytical precision in a previously tared 50–100 mL beaker, are introduced in a vacuum-connected (10^-3^-10^-4^ mm Hg) P_2_O_5_ (phosphorus pentoxide) desiccator, kept at room temperature under the specified conditions until constant weight measured on an analytical balance.

### The amount (content) of water is evaluated with the relation

$${\text{H}}_{2}\text{O}=\frac{{\text{m}}_{1}-{\text{m}}_{2}}{{\text{m}}_{1}}\cdot 100(\%)$$

Where: m_1_: amount of polyethoxylated nonylphenol (n = 3-20), technical product accessed (g); m_2_: amount of polyethoxylated (n = 3-20) and free nonylphenol after drying to constant weight (g).; m_2_: amount of dried polyethoxylated nonylphenol (n = 3-20), technical product accessed in the analysis (g); m_4_: amount of free nonylphenol and/or polyethylene glycol evaluated after the evaporation of the combined ethyl acetate phases (g).; m_2_: amount of dried product accessed in the analysis (g); m_3_: amount of product evaluated after the evaporation of the combined chloroform phases free of polyethyleneglycols (PEG_n_) (g).; m_3_: amount of dried monoderivatized R(NF) heterogeneous (n = 3-20) chain (PEO) free of polyethyleneglycols (PEG_n_) (g); m_4_: amount of monoderivatized R(NF) heterogeneous (n = 3-20) chain (PEO) and free nonylphenol as such in the dried product, free of polyethyleneglycols (PEG_n_) accessed in the evaluation (g).; m_3_: amount of dried polyethoxylated nonylphenol (n = 3-20) (free of polyethyleneglycols) used for analysis (g); m_5_: amount of free nonylphenol (ROH) (NF) extracted from the dried analyzed product (free of polyethyleneglycols) (g).

The dried (hygroscopic) polyethoxylated product is kept further in the desiccator (P_2_O_5_) for further determinations and/or processing.

The final evaluation of the water content is performed in parallel by Karl-Fischer titration.

### Determination (separation) by liquid-liquid extraction of the content of polyethoxylated (n = 3-20) and free nonylphenol in dried technical products

1–2 g dried polyethoxylated (n = 3-20) and free nonylphenol, weighed with analytical precision in a previously tared 50–100 mL beaker, are dissolved in 5–10 mL ethyl acetate, neat, or with a minimal addition of saturated NaCl solution, then transferred quantitatively (by repeated washings with minimal amounts of solvent) into a 250-mL separation funnel. The mixture is shaken vigorously, repeatedly, alternating with stages of decompression of the liquid-liquid extraction system by rotating with 180° and cautiously opening the stopcock of the separation funnel. It is allowed to stand until complete separation of the organic phases from the combined aqueous ones (tendencies of stable emulsification at low oligomerization degrees). We recommend turning the separation funnel in both directions, horizontally on the ring.

The reunited organic phases (ethyl acetate) are dried by filtration over anhydrous Na_2_SO_4_, then evaporated in a vacuum-connected installation (10^-2^-10^-3^ mm Hg).

The product with appearance and fluidity dependent on the oligomerization degree, weighed with analytical precision (m_4_) contains the mixture of polyethoxylated (n = 3-20) and free nonylphenol, and polyethylene glycol from the dried product accessed:

$$\text{polyethoxylated (n=3-20) nonylphenol}=\frac{{\text{m}}_{2}-{\text{m}}_{4}}{{\text{m}}_{2}}\cdot 100(\%)$$

Where: m_1_: amount of polyethoxylated nonylphenol (n = 3-20), technical product accessed (g); m_2_: amount of polyethoxylated (n = 3-20) and free nonylphenol after drying to constant weight (g).; m_2_: amount of dried polyethoxylated nonylphenol (n = 3-20), technical product accessed in the analysis (g); m_4_: amount of free nonylphenol and/or polyethylene glycol evaluated after the evaporation of the combined ethyl acetate phases (g).; m_2_: amount of dried product accessed in the analysis (g); m_3_: amount of product evaluated after the evaporation of the combined chloroform phases free of polyethyleneglycols (PEG_n_) (g).; m_3_: amount of dried monoderivatized R(NF) heterogeneous (n = 3-20) chain (PEO) free of polyethyleneglycols (PEG_n_) (g); m_4_: amount of monoderivatized R(NF) heterogeneous (n = 3-20) chain (PEO) and free nonylphenol as such in the dried product, free of polyethyleneglycols (PEG_n_) accessed in the evaluation (g).; m_3_: amount of dried polyethoxylated nonylphenol (n = 3-20) (free of polyethyleneglycols) used for analysis (g); m_5_: amount of free nonylphenol (ROH) (NF) extracted from the dried analyzed product (free of polyethyleneglycols) (g).

The reunited aqueous phases (saturated NaCl solution) are liquid-liquid extracted repeatedly (2–3 times) with chloroform observing the same working protocol, the organic and inorganic (aqueous) phases, respectively, are completely separated and collected in tared flasks of adequate capacity.

The reunited chloroform phases are filtered (dried) over a layer of anhydrous Na_2_SO_4_ and then evaporated in a previously tared flask in a vacuum-connected installation (10^-2^-10^-3^ mm Hg). The product with appearance and fluidity dependent on the oligomerization degree, weighed with analytical precision (m_3_) contains the free polyethyleneglycols (PEG_n_) in the analyzed product:

$${\text{polyethyleneglycols (PEG}}_{\text{n}})=\frac{{\text{m}}_{3}}{{\text{m}}_{2}}\cdot 100(\%)$$

Where: m_1_: amount of polyethoxylated nonylphenol (n = 3-20), technical product accessed (g); m_2_: amount of polyethoxylated (n = 3-20) and free nonylphenol after drying to constant weight (g).; m_2_: amount of dried polyethoxylated nonylphenol (n = 3-20), technical product accessed in the analysis (g); m_4_: amount of free nonylphenol and/or polyethylene glycol evaluated after the evaporation of the combined ethyl acetate phases (g).; m_2_: amount of dried product accessed in the analysis (g); m_3_: amount of product evaluated after the evaporation of the combined chloroform phases free of polyethyleneglycols (PEG_n_) (g).; m_3_: amount of dried monoderivatized R(NF) heterogeneous (n = 3-20) chain (PEO) free of polyethyleneglycols (PEG_n_) (g); m_4_: amount of monoderivatized R(NF) heterogeneous (n = 3-20) chain (PEO) and free nonylphenol as such in the dried product, free of polyethyleneglycols (PEG_n_) accessed in the evaluation (g).; m_3_: amount of dried polyethoxylated nonylphenol (n = 3-20) (free of polyethyleneglycols) used for analysis (g); m_5_: amount of free nonylphenol (ROH) (NF) extracted from the dried analyzed product (free of polyethyleneglycols) (g).

### Determination (separation) by liquid-liquid extraction of the content of free nonylphenol in polyethoxylated nonylphenol (n = 3-20) chains

1–2 g analyzed mixture weighed with analytical precision in a previously tared 50–100 mL beaker, is dissolved in 5–10 mL 96% ethyl alcohol, then transferred quantitatively (by repeated washings with minimal amounts of solvent) into a 250-mL separation funnel.

A similar volume of petroleum ether (b.p. = 30-60°C) is added, the mixture is shaken vigorously, repeatedly, alternating with stages of decompression of the liquid-liquid extraction system by rotating with 180° and cautiously opening the stopcock of the separation funnel. It is allowed to stand for the complete separation of the organic phases (stable emulsification at low oligomerization degrees). For “breaking” the emulsion and complete separation of the phases the separation funnel is rotated horizontally in both directions on the ring.

The product [the solution previously filtered (dried) over anhydrous Na_2_SO_4_] resulted after vacuum evaporation (10^-2^-10^-3^ mm Hg) of the alcohol phases, with appearance and fluidity dependent on the oligomerization degree, weighed with analytical precision (m_4_) contains the monoderivatized R(NF) heterogeneous (n = 3-20) polyoxyethylene (PEO) chains free of polyethyleneglycols (PEG_n_) in the analyzed (dried) sample:

$$\text{monoderivatized R(NF) heterogeneous (n}=3-20)\;\text{polyoxyethylene (PEO) chains}=\frac{{\text{m}}_{3}}{{\text{m}}_{4}}\cdot 100(\%)$$

Where: m_1_: amount of polyethoxylated nonylphenol (n = 3-20), technical product accessed (g); m_2_: amount of polyethoxylated (n = 3-20) and free nonylphenol after drying to constant weight (g).; m_2_: amount of dried polyethoxylated nonylphenol (n = 3-20), technical product accessed in the analysis (g); m_4_: amount of free nonylphenol and/or polyethylene glycol evaluated after the evaporation of the combined ethyl acetate phases (g).; m_2_: amount of dried product accessed in the analysis (g); m_3_: amount of product evaluated after the evaporation of the combined chloroform phases free of polyethyleneglycols (PEG_n_) (g).; m_3_: amount of dried monoderivatized R(NF) heterogeneous (n = 3-20) chain (PEO) free of polyethyleneglycols (PEG_n_) (g); m_4_: amount of monoderivatized R(NF) heterogeneous (n = 3-20) chain (PEO) and free nonylphenol as such in the dried product, free of polyethyleneglycols (PEG_n_) accessed in the evaluation (g).; m_3_: amount of dried polyethoxylated nonylphenol (n = 3-20) (free of polyethyleneglycols) used for analysis (g); m_5_: amount of free nonylphenol (ROH) (NF) extracted from the dried analyzed product (free of polyethyleneglycols) (g).

The product resulted after the high-vacuum (10^–2^-10^–3^ mm Hg) evaporation of the reunited ether phases with appearance and fluidity dependent on the oligomerization degree, weighed with analytical precision (m_5_) contains the free nonylphenol ROH (R = NF), in the dried technical product analyzed, free of polyethyleneglycols:

$$\text{free nonylphenol R(NH)OH}=\frac{{\text{m}}_{5}}{{\text{m}}_{3}}\cdot 100(\%)$$

where: m_1_: amount of polyethoxylated nonylphenol (n = 3-20), technical product accessed (g); m_2_: amount of polyethoxylated (n = 3-20) and free nonylphenol after drying to constant weight (g).; m_2_: amount of dried polyethoxylated nonylphenol (n = 3-20), technical product accessed in the analysis (g); m_4_: amount of free nonylphenol and/or polyethylene glycol evaluated after the evaporation of the combined ethyl acetate phases (g).; m_2_: amount of dried product accessed in the analysis (g); m_3_: amount of product evaluated after the evaporation of the combined chloroform phases free of polyethyleneglycols (PEG_n_) (g).; m_3_: amount of dried monoderivatized R(NF) heterogeneous (n = 3-20) chain (PEO) free of polyethyleneglycols (PEG_n_) (g); m_4_: amount of monoderivatized R(NF) heterogeneous (n = 3-20) chain (PEO) and free nonylphenol as such in the dried product, free of polyethyleneglycols (PEG_n_) accessed in the evaluation (g).; m_3_: amount of dried polyethoxylated nonylphenol (n = 3-20) (free of polyethyleneglycols) used for analysis (g); m_5_: amount of free nonylphenol (ROH) (NF) extracted from the dried analyzed product (free of polyethyleneglycols) (g).

## Conclusions

Taking over critically from the literature issues related to nonionic soaps in the context of steadily increasing demands of assortment diversification, this work succeeded in presenting preparative and structural aspects related to the synthesis of alkaline and ethanolammonium β-nonylphenoxy (n = 0) propionates and alkaline and ethanolammonium β-nonylphenolpolyethyleneoxy (n = 3-20) propionates, respectively, accessing for the first time a scheme of classical reactions (cyanoethylation, exhaustive acid hydrolysis with DBSH and stoichiometric neutralization), which eliminated previously known and claimed disadvantages.

The direct or indirect evolution of the processing yields was studied as a function of the structural parameters, proper process parameters (temperature, nature and concentration of catalyst, molar ratio of participants), process byproducts.

In the heterogeneous stages of the accessed operation block schemes special attention was given to the role of the interphase transfer catalyst in general, and of the polyoxyethylene chains (PEO) grafted as such and/or derivatized (phase-transfer catalysts PTC) in particular.

Starting from polyethoxylated nonylphenols, technical products contaminated with traces of water, free nonylphenol and/or polyethyleneglycols with various degrees of oligomerization, after specific purification by liquid/liquid and/or solid/liquid extractions in appropriate intervals of temperature, basic catalyst, duration, molar ratio of reactants, acrylic monomer polymerization inhibitor, etc., the influence of these operating parameters on the processing yields was quantified.

The reversibility of cyanoethylation upon prolonged contact of the reaction products with the basic catalyst was also confirmed.

Accessing dodecylbenzenesulphonic acid allowed on the one hand the provision of high yields of exhaustive acid hydrolysis, and on the other hand confirmed the role of acid catalyst in the directed structuring of binary nonionic-anionic colloidal systems for food sanitation.

From the study conducted it can be concluded that structurally the behavior of nonylphenols in the adopted reaction scheme can be divided into two distinct areas: for nonylphenol alone (n = 0) and for polyethoxylated nonylphenols (n = 3-20), respectively.

In the first case the low nucleophilicity of sodium nonylphenoxide due to (−I_s_) and (+E_s_) effects (p-π conjugation) affects cyanoethylation yields negatively. For this reason the presence of PTC_1_-PTC_4_-type phase-transfer catalysts favors the reaction by the coordination of the alkaline cation in the nucleophilic agent, the interphase transfer of the ionic species and catalyst (dodecylbenzenesulphonic acid). The role of micellar catalysis during the exhaustive acid hydrolysis phase cannot be excluded either.

In the second case the normal nucleophilicity specific to alkaline alkoxides (the electronic effects mentioned above being virtually non-existent) provides reaction yields increasing proportionally to the size of the polyoxyethylene chain in its own structure and with the coordination ability of the alkaline cation and/or other ionic species in the reaction medium, respectively.

This work underlies and develops concepts, approaches synergistically, in an inter-and multidisciplinary manner the study of obtaining alkaline and ethanolammonium β-nonylphenolpolyethyleneoxy (n = 3-20) propionates, concerning the interdependence between structure and properties, promotes the idea of design, realization and access to new “highly qualified” nonionic soaps and open new topical thematic areas, with major impact on food sanitation.

The synergistic pairing of individual colloidal competences into a single architecture allowed the monitored modification of the hydrophilic-hydrophobic balance (HLB), correlated with the appropriate changes in surface tension and critical micellar concentration of the new class of nonionic soaps.

Mathematical processing of the experimental data allowed on the one hand graphic evaluation of the development of the processes in a given range of variation of the work parameters, on the other hand the formulation of the closest mathematical relationship which would generalize the studied phenomenon. The high correlation coefficients confirm the "clustered" character of the experimental values recorded.

## Competing interests

The author declares that he has no competing interests.

## Authors' contributions

CJ has performed all the experiments and prepared the final form of the manuscript.

## References

[B1] CAC (Codex Alimentarius Commission) Hazard Analysis and Critical Control Point (HACCP) System and Guidelines for its Applications CAC-RCP 1–1969, rev. 3. Food Hygiene Basic Text. Secretariat of the Joint FAO/WHO Food Standards Programme, FAO 1997 Rome, Italy

[B2] OJL (Official Journal of the European Union) Regulation (EC) No. 852/2004 of the European Parliament and of the Council of 29 April 2004 laying down the hygiene of foodstuffs OJL 139 2004

[B3] Holah JT Stringer M, Dennis C Cleaning and disinfection In Chilled Foods: a Comprehensive Guide 2000 2nd Cambridge: Woodhead Publishing 397 428

[B4] Holah JT Lelieved HLM, Mostert MA, Holah J, White B Cleaning and disinfection Hygiene in Food processing 2003 Cambridge: Woodhead Publishing 235 278

[B5] Sansebastiano G Zoni R Bigliardi L McElhatton A, Marshall RJ Cleaning and Disinfection Procedures in the Food Industry General Aspects and Practical Applications Food Safety A Practical and Case Study Approach 2007 New York: Springer 253 280

[B6] Restle S Cauwet Martjn D Compositions for treating keratinous materials containing a combination of zwitterion polymer and water insoluble non-volatile silicon 2003 Patent Number US 2003/6555100 B1

[B7] Kristensen AB A laundry detergent composition European Patent Office 0508934 A1/1992 1992

[B8] Cripe TA Ofosu-Asante K Alkaline light duty diswashing detergent composition containig an alkyl ethoxy carboxylate surfactant, magnesium ions, chelator and buffer 1994 US 5376310/1994

[B9] Ofosu-Asante K Liquid or gel diswashing detergent containing alkyl ethoxy carboxylate divalent or ions and alkylpolyethoxy polycarboxylate 1998 US 5739092/1998

[B10] Man VFP Alkaline cleaners based on alcohol ethoxy carboxylates 2001 Patent Number US 6274541 B1/2001

[B11] Man VFP Alkaline cleaners based on alcohol ethoxy carboxylates 2002 Patent Number US 6479453 B2/2002

[B12] Leinweber D Dahlmann U Kupfer R Process for the solvent-free preparation of ether carboxylic acids having a low residual salt content 2007 Patent Number US 7208118 B2/2007

[B13] Underwood D Tadrowski TJ Rigley K Cleaner concentrate comprinsing ethanoldiglycine and a tertiary surfactant mixture 2010 Patent Number US 7838484 B2/2010

[B14] Schafer W Schafer R Schafer D Aalbers JG Smid JK Novel polyether carboxylic acid derivatives, as well as their uses 1989 Patent Number US Pat. 4818440/1989

[B15] Haussmann H Scheufeer W Kaupp J Polyoxyalkylene ether acid compounds containing a higher aliphatic group 1939 Patent Number US Pat. 2183853/1939

[B16] Jianu C Colloidal competences of some food cleaning agents based on alkaline and ammonium nonionic soaps J Food Agric Environ 2012 10 1 10 15

[B17] Isa H Inoniya T Karube K Takemoto T Nagayama M Verfahren zur Herstellung von carboxylaten 1973 DE 2.303.829 (A1)

[B18] Rodney MW Thomas AC Light-duty liquid or gel dishwashing detergent composition containing an alkyl ethoxy carboxylate surfactant 1993 Patent Number US Pat. 5230823/1993

[B19] Nenitescu CD Organic Chemistry 1980 8th Bucharest: Didactical & Pedagogical Publishing House in romanian

[B20] Kon-Chang L Preparation of carboxypropylate non-ionic surfactants 1987 Patent Number US Pat. 4692551/1987

[B21] Abe Y Long-Chain Fatty Acids Containing Ether Linkage. I. The antimicrobial and Fungicidal Activities of Some New β-alkyloxypropionic acids and their methyl esters Lipids 1966 1 2 141 145 10.1007/BF02533007 17805669

[B22] Abe Y Osanai S Long-Chain Carboxylic Acids Containing Ether Linkage. III. The Antibacterial and Antifungal Activities of the Amine Salts of Some β-Alkoxypropionic Acids J Am Oil Chem Soc 1969 46 7 365 367 10.1007/BF02636866 5796295

[B23] Abe Y Osanai S Matsumura S Long-Chain Carboxylic Acids Containing Ether Linkage. IV. The Abtibacterial Activities of O-(2-Alkylaminoethyl)-3-oxypropionic and N-(2-Alkyloxyethyl)-3-aminopropionic Acids J Am Oil Chem Soc 1972 49 6 357 360 10.1007/BF02633389 4555486

[B24] Abe Y Osanai S Matsushita T Long-Chain Carboxylic Acids Containing Ether Linkage. V. N-methyl Substituted Glycine-Type Amphoterics Containing Alkoxy Radical J Am Oil Chem Soc 1974 51 9 385 388 10.1007/BF02635012

[B25] Jianu C Nedelea G Lazureanu A Jianu I Adamescu A Diversifying and functional featuring of some peg coacervates as ecological nanocomponents in the soil-plant-man (food consumer) circuit of some nutrients Bull Univ Agric Sci Vet Med 2004 60 507 507

[B26] Jianu C Research concerning the potential of some metal ion complexing additives to improve food value in horticultural raw matter PhD thesis 2006 Banat’s University of Agricultural Sciences and Veterinary Medicine

[B27] Jianu C Butnariu M Cocan I Rinovetz A Bujancă G Jianu I Hydrolysis of β-alkyl (C12H25/C18H37) polyethyleneoxy (n = 3-20) propionitriles in micellar catalysis conditions (I) J Agroalim Proc Technol 2009 15 1 72 78

[B28] Jianu C Traşcă T Riviş A Miscă C Chiş M Jianu I Hydrolysis of β-alkyl (C12H25/C18H37) polyethyleneoxy (n = 3-20) propionitriles in phase transfer catalysis conditions (II) J Agroalim Proc Technol 2009 15 1 79 87

[B29] Jianu C Jianu I Colloidal competences of new tailor-made lipids J Food Agric Environ 2010 8 3&4 148 155

[B30] Izzat R Christensen J Progress in Macrocyclic Chemistry 1979 New York: Wiley & Son Volume I and II

[B31] Schulze K Fettalkoholoxäthylatacetate Seifen, Öle, Fette, Wachse 1975 101 37 41 in german

[B32] Jianu I Aminoethers surfaceactive agents Cationic surfaceactive agents 1984 Polytehnic University: PhD thesis

[B33] Jianu I Beitrage zum kinetischen und thermodynamischen studium der cyanoethylierungs reaktionen höherer alkohole (C10-C18) Proceeding World Surfactants Congress. Surfactants in our Word Today and Tomorrow 1984 II 188 196 n German

[B34] Yanagida S Takahashi K Okahara M Metal-ion complexation of noncyclic poly(oxyethylene) derivates I. Solvent extraction of alkali and alkaline earth metal thiocyanates and iodines Bull Chem Soc Jpn 1977 50 6 1386 1390 10.1246/bcsj.50.1386

[B35] Harvey RD Barlow DJ Drake AF Kudsiova L Lawrence MJ Brain A Heenan RK The effect of electrolyte on the encapsulation efficiency of vesicles formed by the nonionic surfactants (2C18E12) J Colloid Interface Sci 2007 315 2 648 661 10.1016/j.jcis.2007.06.076 17692324

[B36] Yokogama Y Hirajima R Morigaki K Yamaguchi Y Ueda K Alkali-cation affinities of polyoxyethylene dodecylethers and helical conformation of their cationized molecules studied by electrospray mass spectrometry J Am Soc Mass Spectrom 2007 18 11 1914 1920 10.1016/j.jasms.2007.08.004 17881246

[B37] Jackson AT Scrivens JH Williams JP Baker ES Gidden J Bowers MT Microstructural and Conformational studies of polyether copolymers Int J Mass Spectrom 2004 238 3 287 297 10.1016/j.ijms.2004.09.025

[B38] Gidden J Wyttenbach T Jackson AT Scrivens JH Bowers MT Gas-Phase Conformations of Synthetic Poymers: Poly(Ethylene Glycol), Poly(Propylene Glycol) and Poly (Tetramethylene Glycol) J Am Chem Soc 2000 122 19 4692 99 10.1021/ja993096+

[B39] Bogan MJ Agnes GR Poly(ethyleneglycol) Doubly and Singly Cationized by Different Alkali Metal Ions: Relative Cation Affinities and Cation-Dependent Resolution in a Quadruple Ion Trap Mass Spectrometer J Am Soc Mass Spectrom 2002 13 2 177 186 10.1016/S1044-0305(01)00350-6 11838021

[B40] Edwards CL Os NM Polyoxyethylene alcohols Nonionic Surfactants: Organic Chemistry 1998 New York: Marcel Dekker

[B41] Weinheimer RM Varineau PT Os NM Polyoxyethylene alkylphenols Nonionic Surfactants: Organic Chemistry 1998 New York: Marcel Dekker

